# Incidence and determinants of extrapulmonary tuberculosis in Egypt: a retrospective cohort study

**DOI:** 10.1038/s41598-025-95699-z

**Published:** 2025-04-20

**Authors:** Wagdy Amin, Rasha Ashmawy, Sarah Assem Ibrahim, Yousra A. El-Maradny, Neamat Hamdy Elsawy, Shaimaa Abdallah Gebili, Nahla Gamaleldin, Ramy Mohamed Ghazy

**Affiliations:** 1 General Administration of Chest Diseases, MoHP, Cairo, Egypt; 2 Clinical Research Department, Maamora Chest Hospital, MoHP, Alexandria, Egypt; 3 PhD Candidate at Medical Research Institute, Alexandria University, Alexandria, Egypt; 4 Department of Biostatistics and Demography, Faculty of Graduate Studies for Statistical Research, Cairo University, Giza, Egypt; 5 Pharmaceutical and Fermentation Industries Development Center, City of Scientific Research and Technological Applications (SRTA-City), New Borg EL-Arab, Alexandria, 21934 Egypt; 6 Department of Clinical Research, Fowa Central Hospital, MoHP, Kafr El Sheikh, Egypt; 7 Clinical Research Administration, Directorate of Health Affairs, MoHP, Fayoum, Egypt; 8 Department of Community Medicine, Faculty of Medicine, Alexandria University, Alexandria, Egypt; 9 Tropical Health Department, High Institute of Public Health, Alexandria University, Alexandria, Egypt

**Keywords:** Pulmonary tuberculosis, Extrapulmonary tuberculosis, Human immunodeficiency virus, Co-infection, Hepatitis C, Diseases, Infectious diseases, Bacterial infection, HIV infections, Tuberculosis

## Abstract

Tuberculosis (TB) is a significant global public health concern. The incidence of extrapulmonary tuberculosis (EPTB) is increasing; however, comprehensive data on its epidemiological and clinical characteristics remain limited, especially among populations who are co-infected with human immunodeficiency virus (HIV) or hepatitis C virus (HCV). This study aimed to assess the incidence and predictors of EPTB in patients co-infected with HIV or HCV in Egypt. We conducted a retrospective cohort study on patients infected with TB who are treated in Egyptian chest hospitals from January 1 to December 31, 2023. Patients were categorized into pulmonary TB (PTB) and EPTB. Clinical data, including HIV or HCV co-infection status, were analyzed to identify risk factors and comorbidities associated with EPTB. Multilevel logistic regression was employed to examine predictors of EPTB. Among 7,245 TB patients, 42.5% were diagnosed with EPTB. Determinant of EPTB were HIV-positive (OR = 0.46, 95% CI: 0.30–0.71, *p* < 0.001), being male (OR = 0.31, 95% CI: 0.27–0.35, *p* < 0.001 ), age (particularly children under 5 years) (OR = 4.75, 95% CI: 2.29–9.84, *p* < 0.001 ), urban residency (OR = 1.05, 95% CI: 0.87–1.27, *p* < 0.05), and comorbidities (OR = 0.59, 95% CI: 0.35–0.98, *p* < 0.05). The most common sites for EPTB were the lymph nodes (27.10%) and pleural cavity/effusion (24.60%). EPTB represents a substantial proportion of TB cases in Egypt, particularly among younger individuals and females. Despite the low percentage of HIV or HCV co-infection in EPTB cases, further analysis and diagnostic testing of undiagnosed patients are required. These findings underscore the need for targeted interventions and comprehensive care models for TB patients, especially in the context of HIV co-infection.

## Introduction

Tuberculosis (TB) is a communicable disease caused by *Mycobacterium tuberculosis *(MTB) and is one of the leading causes of death worldwide. By 2023, TB likely regained its position as the leading cause of death globally from a single infectious agent, after being surpassed for three years by coronavirus disease 2019 (COVID-19). TB causes nearly twice as many deaths as human immunodeficiency virus/ acquired immunodeficiency syndrome (HIV/AIDS)^1^. Globally, an estimated 10.8 million TB cases occurred in 2023, and this number has been increasing since 2021^2^. Around one-fourth (24%) of TB cases are diagnosed in Africa, where TB is endemic. In Egypt, the incidence rate of active TB was 10 per 100,000 people in 2021^3,4^.

In 2022, despite the availability of effective tools for prevention, diagnosis, and treatment, the estimated number of deaths due to viral hepatitis increased from 1.1 million in 2019 to 1.3 million in 2022^5^. Chronic hepatitis C virus (HCV) infection is a major cause of morbidity and mortality worldwide. Globally, an estimated 50 million people are infected with HCV, with the vast majority remaining undiagnosed and untreated and 240,000 deaths annually^6^. Egypt has made significant progress in reducing HCV prevalence, shifting from one of the highest rates globally to one of the lowest. Over a little more than a decade, the prevalence of HCV in Egypt decreased from 10 to 0.38%^7^. In 2022, the total number of HCV infections in Egypt was 484,523 patients^6^. The burden of disease for both TB and HCV is substantial, particularly in low- and middle-income countries (LMICs)^8,9^. Although data are limited, a systematic review conducted from January 2000 to March 2018 reported that the overall prevalence of HCV infection in patients with TB was 7% [95% confidence interval (CI): 6–9]^10^. Additionally, there is some geographical overlap in regions with a high burden of both TB and HCV, where high rates of incarceration and injection drug use exacerbate the epidemics^11^.

As of the end of 2022, approximately 39.0 million individuals [33.1–45.7 million] were living with HIV^12^. The incidence of HIV infections decreased slightly from 1.5 million in 2020 to 1.3 million in 2022^5^. Sub-Saharan Africa has the highest TB burden, fueled primarily by high prevalence of HIV infection, followed by Southeast Asia. TB and HIV frequently accelerate each other’s progression, with one disease enhancing the effect of the other. For HIV-infected patients, the risk of developing TB disease is approximately 22 times higher than for people with a protective immune response^13^. TB remains a significant cause of death among people living with HIV. In 2022, the estimated number of deaths caused by TB among people with HIV was 167,000^14^.

Pulmonary tuberculosis (PTB) remains the most common form of TB while,  the proportion of extrapulmonary tuberculosis (EPTB) cases is also significantly high^15^. EPTB is defined as when the disease disseminates to other organs, such as the lymph nodes, central nervous system, gastrointestinal tract (GIT), pleura, genitourinary tract, bones, or other organs^15^. The complexities in diagnosis and management of EPTB hinder physicians’ ability to evaluate and treat these patients promptly^16^. In 2021, the World Health Organization (WHO) reported that EPTB represents 17% of total TB cases worldwide. In the Eastern Mediterranean Region, the prevalence of EPTB exceeds the global prevalence, accounting for 23% of total TB cases^17^. Factors influencing the prevalence of EPTB differ across countries. These include age, gender, socioeconomic status, diabetes mellitus, immunosuppression, malnutrition, and having HIV/AIDS. These factors primarily increase susceptibility to both TB infection and its extrapulmonary manifestations^15^. This study aimed to calculate the incidence of EPTB in Egypt and investigate its predictors, including HIV or HCV co-infections, as well as patients’ sociodemographic and clinical characteristics.

## Methodology

### Study design and settings

This multicenter retrospective cohort study was conducted in chest hospitals under the Ministry of Health and Population (MoHP) across 25 governorates of Egypt. This study aimed to assess the incidence and predictors of EPTB among TB patients with and without HIV or HCV co-infections, using the national TB registries. After obtaining ethical approval, data were collected from TB patient files maintained in hospital databases between January 1, 2023, and December 31, 2023.

### Study population and clinical measurements

The study included all patient files documenting confirmed TB cases, irrespective of age or gender. Both hospitalized and outpatient cases from MoHP chest hospitals were included. Files from private clinics, non-MoHP hospitals, or those with incomplete data were excluded.

### Patient diagnosis and confirmation criteria

TB cases were diagnosed in accordance with the Egyptian protocol for TB diagnosis, using a combination of clinical examinations and laboratory findings, including sputum microscopy for acid-fast bacilli (AFB), solid conventional culture (Lowenstein-Jansen medium) for smear-negative PTB or EPTB, and molecular testing via GeneXpert MTB/RIF. For EPTB, diagnostic imaging studies such as abdominal ultrasound (for abdominal TB), echocardiography (for TB pericarditis), spinal magnetic resonance imaging (MRI) for spinal TB, and other radiological modalities were used. EPTB was further confirmed through tissue biopsies, histopathological examination, and fluid analyses, such as cerebrospinal fluid testing with GeneXpert MTB/RIF. The WHO strongly recommends the use of the lateral flow urine lipoarabinomannan (LF-LAM) assay to support the diagnosis of PTB and EPTB in HIV-positive adults and children^18^. According to the Egyptian protocol for screening of HIV^19 ^and HCV^20^, both viruses are initially screened using rapid diagnostic tests (RDTs). For HCV, a positive result is confirmed by polymerase chain reaction (PCR) testing for HCV RNA. In case of HIV, enzyme-linked immunosorbent assay (ELISA) testing is used for confirmation. 

### Data collection

We extracted patient demographic, clinical, and laboratory data from hospital databases and clinical notes, and were recorded in Excel spreadsheets. To ensure data accuracy, we cleaned the dataset through cross-tabulation to address missing entries and frequency checks to avoid duplication. Collected data included sociodemographic details (age, sex, weight, occupation, nationality, and address), classification of TB as PTB or EPTB, diagnostic methods, and imaging findings. Laboratory results included sputum AFB, GeneXpert MTB/RIF, histopathological findings, and HIV and HCV diagnostic tests’ results.

### Outcomes

The primary objective of this study was to determine the incidence of EPTB among TB patients with and without HIV or HCV co-infections. Secondary objective included studying the predictors and risk factors contributing to EPTB patients including HIV or HCV coinfection.

### Statistical analysis

The analysis used a chi-squared test of independence with Phi and Cramer’s V to examine associations between TB patients’ demographic and clinical characteristics (e.g., gender, age, residence, HCV and HIV status, and comorbidities) and type of TB (PTB vs. EPTB). A Z-test compared the proportions of EPTB diagnoses between HIV-negative and HIV-positive patients. A multilevel logistic model, implemented with the R package lme4 using Maximum Likelihood with Laplace Approximation, evaluated the impact of explanatory variables on the probability of EPTB^21^. The model accounted for variations between Egyptian governorates by incorporating cluster-specific residuals.

Patient-related factors (gender, age, comorbidities, and HCV and HIV status) and governorate-level factors (place of residence, human development index (HDI) were stratified into two levels. Initially, a model with only a fixed intercept was tested, followed by the inclusion of random effects, assessed using a likelihood ratio test. The final model included random effects for both intercepts and slopes of independent variables across governorates. The intraclass coefficient (ICC) and the design effect, (design effect = 1+ (average group size − 1) *ICC)^22^, were calculated to assess the significance of the multilevel model. Model fit was evaluated using the akaike information criterion (AIC), bayesian information criterion (BIC), and likelihood ratio tests, balancing fit quality against model complexity. Model significance was evaluated through the ICC and the design effect, The HDI, as defined by the United Nations Development Program (UNDP), is a summary measure of human development based on indices for education, health, and standard of living. It is categorized into four levels: very high (HDI ≥ 0.800), high (HDI 0.700–0.799), medium (HDI 0.550–0.699), and low (HDI < 0.550)^23^.

## Results

### Demographics and characteristics of TB patients

**Fig. 1:**
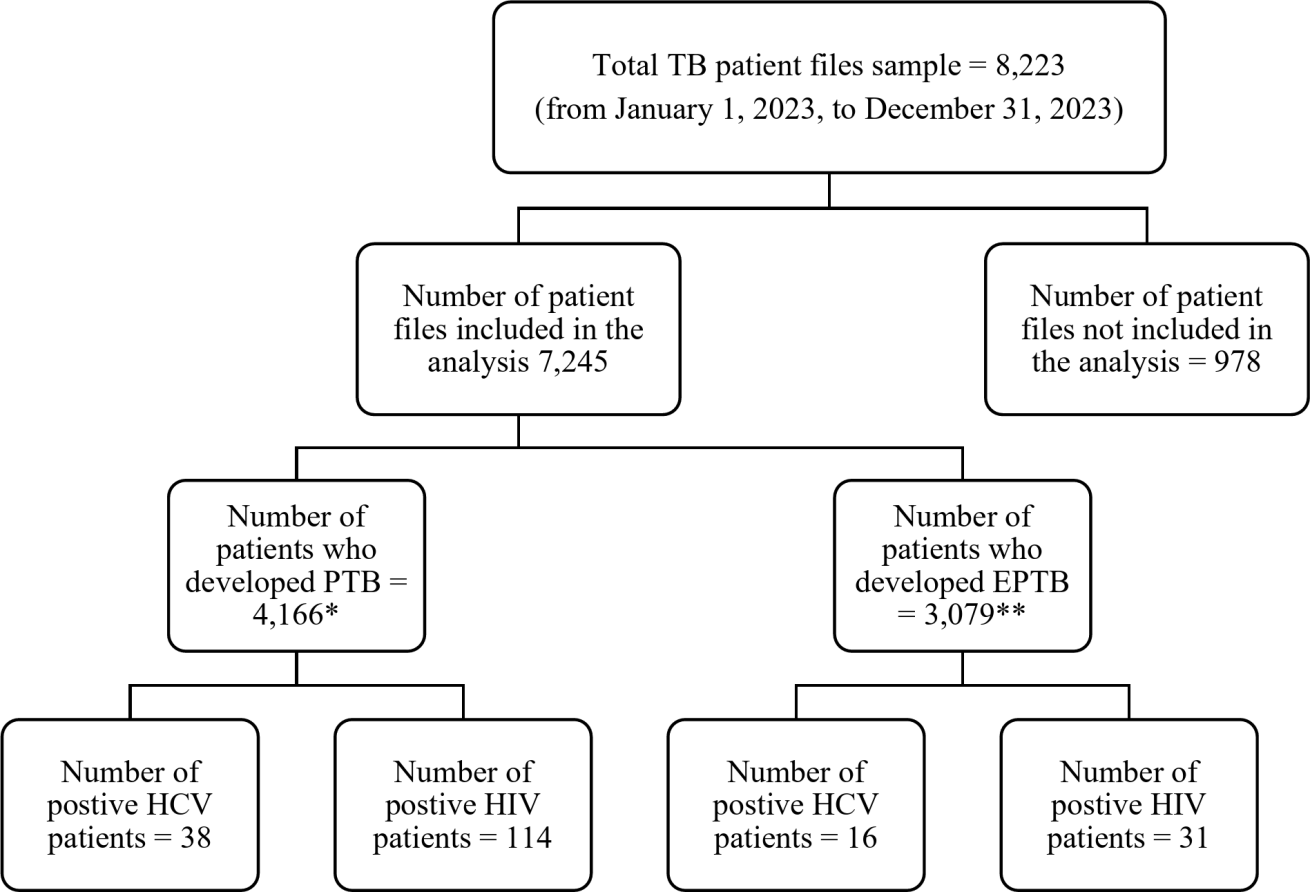
Flowchart showing the assessment of EPTB incidence among TB patients with and without HIV or HCV co-infections in Egypt from January 1 to December 31, 2023. * PTB with both HIV and HCV = 12 and ** EPTB with both HIV and HCV = 2. (N.B. HCV test not performed for 6,184 patients, and HIV test not performed for 2,846 patients)

A total of 8,223 TB patient records were reviewed, with 978 records excluded from the analysis due to incomplete records or diagnoses made outside of MoHP hospitals and clinics.  Among the analyzed patients 7245, 33.5% were aged > 15–30 years, 66.8% were male, and 74.3% were residents in urban areas. The vast majority (98.7%) had no documented comorbidities, and 42.5% presented with EPTB. Among the patients tested for HIV or HCV coinfection, 2.0% and 0.74% tested positive, respectively. Furthermore, when categorized by HDI, the majority (84.8%) of individuals resided in high-HDI areas, followed by 14.3% in medium-HDI regions, and only about 0.9% in very high-HDI locations, as shown in Fig. 1 and Table 1.

**Table 1:** Characteristics of TB patients and children living in 25 Egyptian governorates.

Variables (*N* = 7,245)	Categories	*N* (%)
Gender	Male	4,838 (66.8)
Age groups in years	Less than five	112 (1.5)
	5–15	328 (4.5)
	> 15–30	2,426 (33.5)
	> 30–45	2,268 (31.3)
	> 45–60	1,223 (16.9)
	> 60	888 (12.3)
Residence place	Rural	1,862 (25.7)
	Urban	5,383 (74.3)
TB clinical presentation	EPTB	3,079 (42.5)
	PTB	4,166 (57.5)
Comorbidities*	None	7,149 (98.7)
	One or more	96 (1.3)
HIV status	Positive	145 (2.0)
	Negative	4,254 (58.72)
	Not performed	2,846 (39.28)
HCV status	Positive	54 (0.74)
	Negative	1,007 (13.90)
	Not performed	6,184 (85.36)
HDI categories	Very high	64 (0.9)
	High	6,147 (84.8)
	Medium	1,034 (14.3)

PTB = pulmonary tuberculosis; EPTB = extrapulmonary tuberculosis; HDI = Human Development Index

*Comorbidities include cancer, cardiac disease, chronic obstructive pulmonary disease, diabetes mellitus, epilepsy, hemophilia, hepatic disease, hypertension, neurological disease, renal disease, and rheumatoid conditions.

**Fig. 2:**
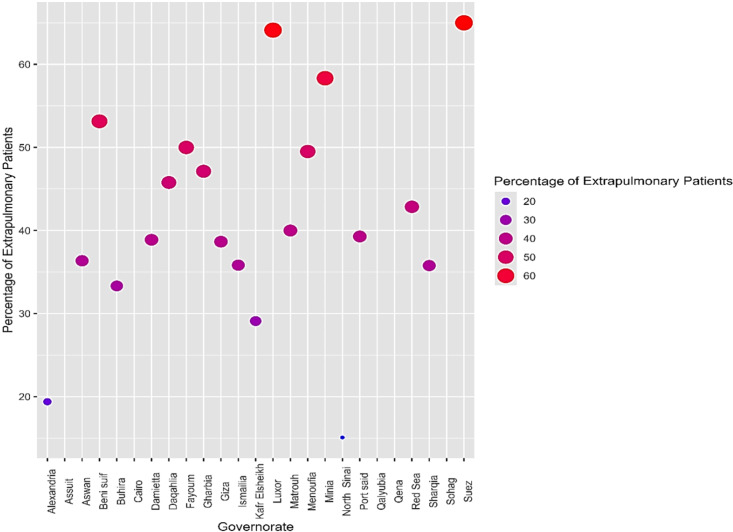
A bubble plot showing the percentage of EPTB patients across 25 Egyptian governorates. Each bubble represents the percentage of EPTB in a specific governorate, with the size and color intensity of the bubbles corresponding to the percentage of affected patients.

Figure 2 illustrates the percentage of EPTB across Egyptian governorates, highlighting a high prevalence in Suez and Luxor, where over 60% of patients had EPTB. This is followed by Minia and Beni Suef, with approximately 50% of patients affected. In contrast, the lowest percentages were observed in North Sinai and Alexandria, where less than 20% of patients had EPTB.

**Fig. 3:**
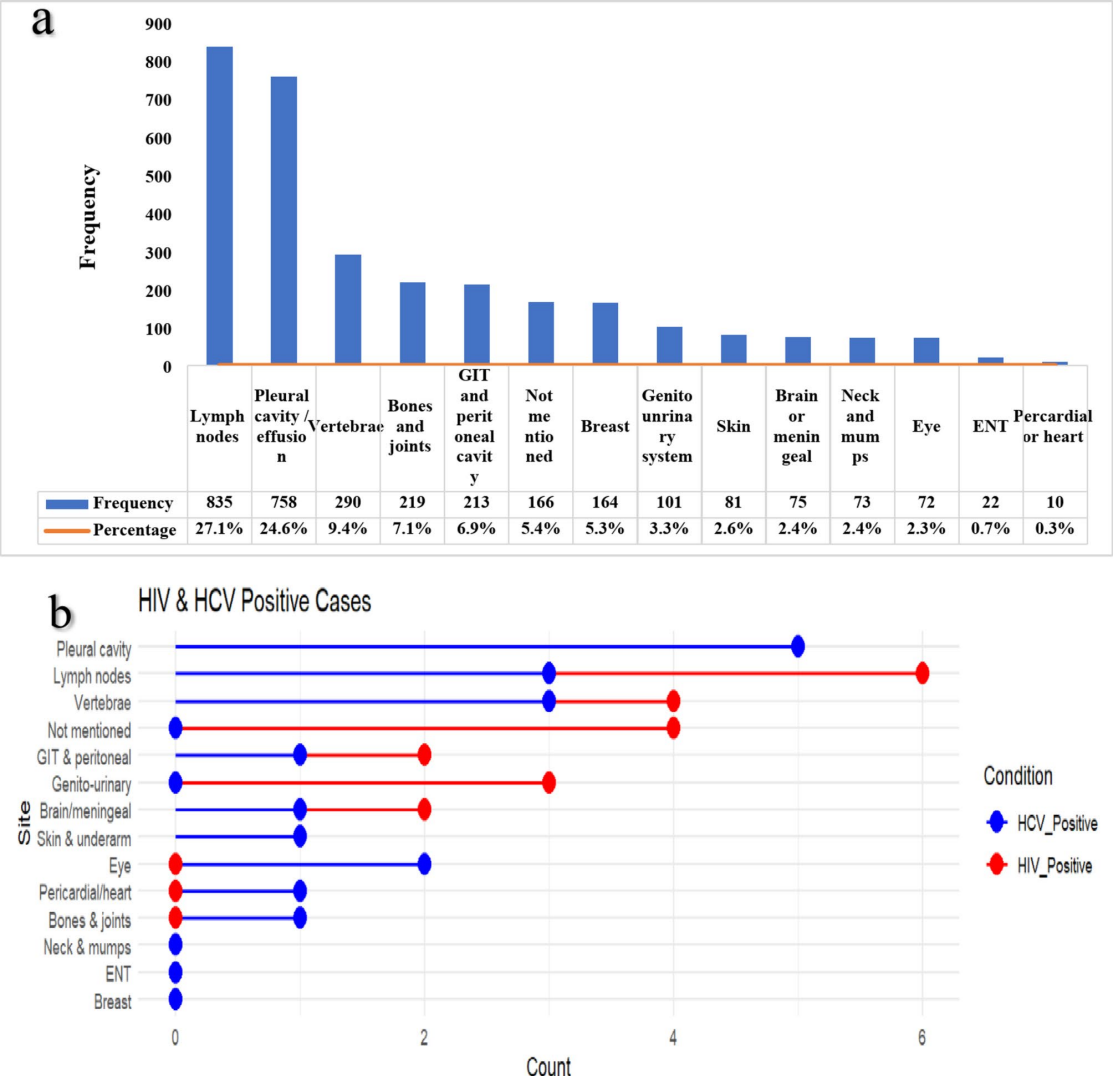
EPTB site distribution among studied populations, these numbers were mutually exclusive. (a) Distribution of EPTB cases across anatomical sites. (b) Distribution of HIV and HCV-positive EPTB cases. *GIT*: Gastrointestinal tract; *ENT*: Ear, nose, and throat.

Figure 3a illustrates the distribution of EPTB cases across various anatomical sites, with the lymph nodes (27.1%) and pleural cavity/effusion (24.6%) being the most frequently affected sites. Other commonly impacted sites included the vertebrae (9.4%), bones and joints (7.1%), and the gastrointestinal and peritoneal cavity (6.9%). Less commonly affected sites included breast, genitourinary system, skin, brain or meninges, neck and salivary glands, and eye, while cases involving the ear, nose, throat, and pericardium/heart were rare.

Figure 3b further categorizes these EPTB cases based on HIV and HCV positivity, illustrating the distribution of infections across different anatomical sites. HCV-positive cases were more frequently observed in the pleural cavity and lymph nodes, with up to 6 cases in some locations, whereas HIV-positive cases were distributed across multiple sites, including the pericardium/heart (2 cases), bones & joints (2 cases), and skin & underarm (2 cases). Notably, certain sites, such as the brain/meninges (3 cases), genitourinary system (2 cases), and eye (2 cases), showed a higher prevalence of HCV-positive cases compared to HIV-positive cases.

### Clinical and epidemiological associations with EPTB and PTB

Table 2 presents the distribution of EPTB and PTB cases across various demographic and clinical variables. HIV status showed a notable relationship, with HIV-positive individuals having a higher percentage of PTB (78.6%) compared to EPTB (21.4%), indicating a significant association (*p* < 0.001). Gender analysis revealed that females had a significantly higher percentage of EPTB (62.2%) than males (37.8%) with significant association (*p* < 0.001). Age was a significant factor, with younger individuals showing higher EPTB percentage, decreasing with age (*p* < 0.001). Urban residents had a higher percentage of PTB (59.7%) than in EPTB (40.3%), with a significant association (*p* < 0.001). Comorbidities also affected TB clinical presentation, with a higher percentage of PTB observed in individuals with one or more comorbidities (69.8%) compared to those with EPTB (29%, *p* = 0.017). Additionally, the HDI was significantly associated with TB type, with lower rates of EPTB in higher HDI categories (*p* < 0.001). In contrast, HCV status did not show a significant difference in TB percentage between HCV-positive and HCV-negative individuals (*p* = 0.601).

**Table 2:** TB diagnosis by patient characteristics.

Label	Levels	EPTB *n* (%)	PTB *n* (%)	*p* value
HIV status	Negative	1878 (44.1)	2376 (55.9)	< 0.001
	Positive	31 (21.4)	114 (78.6)	
Gender	Female	1498 (62.2)	909 (37.8)	< 0.001
	Male	1594 (32.9)	3244 (67.1)	
Age in year	Less than five	81 (72.3)	31 (27.7)	< 0.001
	5 − 15	226 (68.9)	102 (31.1)	
	> 15–30	970 (40.0)	1456 (60.0)	
	> 30–45	902 (39.8)	1366 (60.2)	
	> 45–60	526 (43.0)	697 (57.0)	
	> 60	388 (43.7)	500 (56.3)	
Residence	Rural	910 (48.9)	952 (51.1)	< 0.001
	Urban	2169 (40.3)	3214 (59.7)	
Comorbidities	None	3063 (42.8)	4086 (57.2)	0.017
	One or more	29 (30.2)	67 (69.8)	
HCV status	Negative	343 (34.1)	664 (65.9)	0.601
	Positive	16 (29.6)	38 (70.4)	
HDI	Medium	599 (57.9)	435 (42.1)	< 0.001
	High	2458 (40.0)	3689 (60.0)	
	Very high	37 (57.8)	27 (42.2)	

### Multilevel logistic model results

In the null model with only an intercept, a likelihood ratio test comparing models with and without a random component revealed a significant difference (χ² = 369.66, df = 1, *p* = 0.0001). The ICC was 0.010, and the design effect was 12.9, indicating the necessity of a multilevel model with a random intercept, reflecting variability among governorates relative to the total variance. Table 3 shows the odds ratio (OR) for EPTB compared to PTB as 0.93, with a fixed intercept of −0.07, corresponding to a 48% probability of EPTB. The within-group variance in the logit of the odds is 3.29 (σ²), and the random intercept variance is 0.36 (σ00). The data from 25 Egyptian Governorates, totaling 4374 observations, demonstrated considerable variability in random effects, with a governorate-level variance (σ00) of 0.44 and an ICC of 0.13, indicating that 13% of the variance in TB type is explained by differences between governorates.

Adding random slopes for significant variables did not produce substantial results. HIV-positive individuals were less likely to have EPTB (OR = 0.46, 95% CI: 0.30–0.71, *p* < 0.001). Males had a 69% lower likelihood of EPTB compared with females (OR = 0.31, 95% CI: 0.27–0.35, *p* < 0.001). Younger age significantly increased the odds of EPTB compared to the reference category (30–44 years), particularly in children under five years (OR = 4.75, 95% CI: 2.29–9.84, *p* < 0.001) and those aged 5–15 years (OR = 2.99, 95% CI: 2.07–4.33, *p* < 0.001). Comorbidities were associated with a 41% reduction in the odds of EPTB (OR = 0.59, 95% CI: 0.35–0.98, *p* < 0.05).

Contextual variables, including place of residence (OR = 1.05, 95% CI: 0.87–1.27, *p* > 0.05) and HDI categories; high HDI (OR = 1.69, 95% CI: 0.86–3.3, *p* > 0.05), and very high HDI (OR = 1.29, 95% CI:0.64–2.64, *p* > 0.05) did not significantly increase the odds of having EPTB. The marginal R² indicated that fixed effects explained 11% of the variability, while the conditional R², incorporating random effects, explained 22%.

**Fig. 4:**
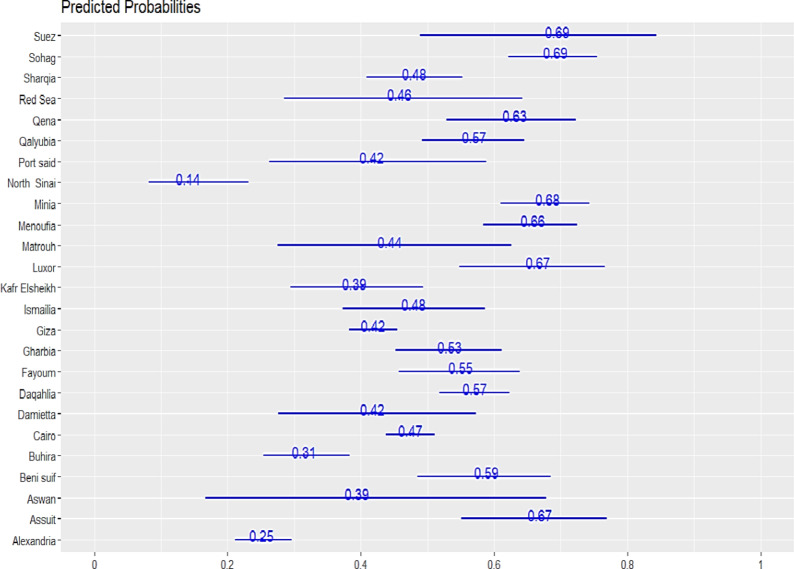
The predicted probabilities of EPTB diagnosis patients with random effect intercept.

Figure 4 highlights the predicted probabilities of EPTB across various governorates. The highest probabilities were observed in Suez, Sohag, Minia, Luxor, and Assiut, with predicted probabilities of 0.69, 0.69, 0.68, 0.67, and 0.67, respectively. Other governorates, such as Red Sea, Qena, and Gharbia, also showed relatively high probabilities (0.63, 0.57, and 0.53). In contrast, lower probabilities were observed in governorates like Alexandria (0.25), Buhira (0.31), and North Sinai (0.14). This variability suggests that geographical factors may influence the likelihood of EPTB.

**Table 3:** The multilevel model with random intercept (EPTB patients).

	EPTB	
Predictors	Odds Ratios	CI
(Intercept)	1.08	0.56–2.10
HIV status [Negative]	Reference	Reference
HIV status [Positive]	0.46 ^***^	0.30–0.71
Gender [Female]	Reference	Reference
Gender [Male]	0.31 ^***^	0.27–0.35
Less than five	4.75 ^***^	2.29–9.84
5–15 Years	2.99 ^***^	2.07–4.33
> 15–30 Years	0.96	0.82–1.12
> 30–35 years	Reference	Reference
> 45–60 Years	0.99	0.81–1.20
> 60 Years	0.92	0.73–1.15
Residence [Rural]	Reference	Reference
Residence [Urban]	1.05	0.87–1.27
Comorbidities [None]	Reference	Reference
Comorbidities [One or more]	0.59 ^*^	0.35–0.98
HDI category [Medium]	Reference	Reference
HDI category [High]	1.69	0.86–3.30
HDI category [Very high]	1.29	0.64–2.64
Random Effects		
σ^2^	3.29	
τ_00 Governorate_	0.47	
ICC	0.13	
N _Governorate_	25	
Observations	4374	
Marginal R^2^ / Conditional R^2^	0.106 / 0.218	

* *p* < 0.05 ** *p* < 0.01 *** *p* < 0.001

## Discussion

Globally, TB is one of the major causes of morbidity and mortality. EPTB constitutes about 15–20% of all TB cases, but this figure rises to 50% among HIV-coinfected patients. However, little is known about the burden and risk factors associated with EPTB in Africa. This study describes the incidence and predictors of EPTB patients who presented to MoHP hospitals across Egypt from January 1, 2023, to December 31, 2023. Data were collected and analyzed for 7,245 patients.

**Incidence of EPTB**: Among the included patients, 57.5% were diagnosed with PTB, and 42.5% with EPTB. The most frequent site of EPTB cases was found to be the lymph nodes (27.10%), followed by the pleural cavity/effusion (24.60%). The remaining EPTB cases were distributed among the vertebrae, bones and joints, GIT, peritoneal cavity, and other sites. These results were aligned with most studies on EPTB^24,25^. This distribution may reflect the focus of control programs primarily on reducing contagious PTB cases, potentially leading to less effective management of EPTB^24^. Moreover, factors such as improved diagnostic methods and enhanced reporting systems for EPTB could also explain the increase in EPTB cases. The incidence of EPTB in a low-TB prevalence setting has been documented at 39.3%^24^for example, in Saudi Arabia, the EPTB percentage reached 57.5%^25^. Shifts in demographics, particularly the growth of populations with a higher predisposition to EPTB, may be a key factor in sustaining these rates^15,26^. For instance, the European Union saw an increase in EPTB cases from 16.4% in 2002 to 22.4% in 2011^27^. In the United States, data from the Centers for Disease Control and Prevention (CDC) shows that the overall decline in TB cases since 1953 is almost exclusively due to the reduction of PTB cases, with little to no decrease in EPTB^28^. This high proportion of EPTB emphasizes the need for further investigation into its determinants, to identify high-risk patients and improve TB management and prognosis.

**Gender and EPTB**: Most of the patients in this study were males aged 15–45, aligning with previous reports identifying this gender and age group as highly affected by TB^15,29,30^. However, in the case of EPTB, the reverse was true^15^. This gender disparity may be attributed to hormonal factors, smoking, and differential exposure to TB, highlighting the need for more targeted TB control programs for these age groups^31,32^. Further analysis of gender differences suggests that lower medical consultation rates among females, potentially due to lower education levels, and greater male exposure to outdoor activities, could explain the higher TB incidence in males^32^. However, a multicenter study found that EPTB was more common in females (30.5%) than in males (27.9%)^33^, a result we also observed in our study. These findings underscore the need for enhanced maternal and neonatal health programs to ensure better access to TB care for women.

**Age and EPTB**: A notable finding in our study is the high incidence of EPTB among children under 15 years of age. Young children, especially those in their first year of life, are at greater risk for severe disseminated TB due to lymphohematogenous spread. Therefore, it is crucial to maintain a high level of suspicion for EPTB involvement when diagnosing TB in this population^34^. We also observed a decline in EPTB incidence with advancing age, contrasting with the increase in PTB incidence in older populations. This may be explained by age-related immune system changes. Studies have shown a functional decline in monocytes and macrophages with age, and a higher production of proinflammatory cytokines in older individuals^35^. However, some studies have reported the highest EPTB risk in individuals over 65 years of age^15^. Further research is needed to further understand these age-related trends and the underlying mechanisms.

**Geographic location and EPTB**: Geographically, our findings are consistent with a retrospective study in Giza that reported a higher incidence of TB in urban areas, with PTB being more common than EPTB in these regions. Contributing factors include overcrowding and air pollution, particularly in urban slums^32^. Conversely, studies from Sohag and El-Behira Governorates, Egypt reported higher TB incidence in rural areas^36,37^. In our study, the highest EPTB incidence was recorded in Cairo, followed by Giza, Sohag, and Assiut with no effect of urban or rural residence. These findings indicate that, while geographic location increases TB burden, other factors such as healthcare availability, population density, and environmental conditions may be more important in EPTB distribution.

**HIV and EPTB**: Despite Egypt’s low HIV prevalence (< 0.1%) among the general population^38^, studies have reported higher HIV co-infection rates among TB patients, with a systematic review estimating it at 54%^30^. In our study, only 2.0% of TB patients were HIV-positive, with PTB being more common than EPTB in this group. This contrasts with findings from Southwest China, where HIV was strongly linked to EPTB^39^, and from sub-Saharan Africa, where HIV prevalence in EPTB patients ranged from 6.4 to 36.8%, likely due to higher rates of advanced HIV infection (65%) and co-factors such as undernutrition^40^. A retrospective study in Uganda found an even stronger association, with 100% of patients who had both PTB and EPTB also being HIV-positive ^41^. Our results align with studies in the Eastern Mediterranean Region, where PTB was more frequent (73.8%) than EPTB (26.2%) among HIV-TB co-infected patients, particularly in those with CD4 + counts > 200 cells/µl, suggesting that preserved immune function favors PTB presentation^42^. Similarly, studies by Shastri et al. (73.2% PTB among 6,480 HIV-TB patients)^43^, Tabarsi et al. (86% PTB)^44^, and Kamath et al.^45^ (58.8% PTB) reported similar finding. Our findings are further supported by a study conducted in South India on 80 HIV-TB co-infected patients, in which 73.8% had PTB and 26.2% had EPTB, with a mean CD4 + T lymphocyte count of 164.7 cells/µl^46^. However, other research, such as that by Mohan et al.^42^, found a higher proportion of EPTB cases (61%) compared to PTB (39%) indicating variability across different populations. Additionally, Rolo et al.^47 ^reported no significant difference in HIV prevalence between PTB and EPTB cases. The low overall HIV prevalence in Egypt and the absence of a high proportion of advanced HIV cases may explain why our findings differ from regions where HIV is a major driver of EPTB. Finally, it is worth noting that patients diagnosed with positive HIV accounts for 2% of the sample, a low percentage of successes in the binary independent variable can be misleading in ordinary ways of estimation. However, we repeated the regression model with the bootstrapping estimation method. Yet, the results of bootstrapping estimation ensured the previous results of odds ratio with 0.49 and 95% confidence interval (0.3–0.7)^21^. Regarding HCV, we did not find a significant association between HCV and TB clinical presentation, as the proportion of HCV cases was too low to provide meaningful data interpretation.

**Comorbidities and EPTB**: Additionally, we found that EPTB incidence was lower in patients with comorbidities. This observation aligns with findings from China^35^, but contrasts with the United States study where diabetes increased the risk of EPTB^48^. Another study found that diabetes mellitus was more prevalent among PTB patients (27.6%) compared to EPTB cases (12.6%) (*p*< 0.001)^39^. These results highlight the need for early diagnosis and management of comorbidities to improve TB treatment outcomes and further explore the association between TB clinical presentation and comorbidities.

**HDI and EPTB**: The data on HDI provides valuable insight into the socioeconomic factors influencing the clinical presentaion of TB (PTB vs. EPTB). It suggests that TB remains a significant public health concern, even in regions characterized by high human development. Although most research emphasizes a higher incidence of TB in areas with low HDI^49^, some studies demonstrate an increase in TB incidence in high-HDI regions^50,51^. This rise is influenced by two opposing factors: high-income levels are typically associated with improved health outcomes, but correlate with higher population density, which facilitates TB transmission. The persistence of TB in high-HDI areas may also be attributed to rapid urbanization, dense populations, and efficient healthcare reporting systems. Moreover, the higher detection rates of EPTB in high-HDI regions may reflect the availability of well-trained practitioners and advanced diagnostic tools, which can identify more complex forms of TB that might be underdiagnosed in lower-HDI settings. However, small sample sizes (< 70 cases) in very high-HDI governorates cause fluctuations in EPTB proportions, making them appear exaggerated compared to larger datasets. Furthermore, many TB cases in these regions originate from migrants from lower-HDI areas, where delayed TB diagnosis is common. Migrants with latent TB infections may develop EPTB instead of PTB due to stress, malnutrition, or immunosuppression, further influencing TB patterns in high-HDI governorates^52^.

## Strengths and limitations

The study uses a national dataset of 7,245 patients in Egypt to analyze the incidence of EPTB cases in public sector healthcare. It explored the clinical interaction between TB clinical presentation (PTB vs EPTB) and different patients’ characteristics using multilevel logistic regression models. However, as a retrospective cohort study, the research is subject to biases such as missing data and the incomplete patient files. A substantial proportion of patients were not tested for HCV (85.36%), limiting the analysis of the co-infection effect. Although the study covered a broad geographic area, differences in healthcare access and diagnostic capabilities between urban and rural regions may affect the results. The lack of detailed clinical information on TB treatment regimens, adherence, and long-term outcomes restricts the ability to fully assess the impact of co-infections on treatment efficacy and prognosis. The data on comorbidities collected from the patient files may be incomplete or underdiagnosed, as no testing for patient comorbidities was conducted. Finally, the study was limited to data and patients from the Egyptian MoHP chest hospitals, excluding cases managed in private clinics or non-MoHP facilities.

## Conclusions

This study examines the incidence and predictors of EPTB among patients with TB in Egypt, focusing on specific demographics and regions. It was found that females and young children were at a higher risk of developing EPTB. Interestingly, HIV positivity and having comorbidities reduced the risk of acquiring EPTB compared to others. The most common sites for EPTB were lymph nodes and pleural cavity. The large proportion of TB patients who were not tested for HIV and HCV highlights the need to improve routine screening. Expanding HIV and HCV testing in TB programs will support earlier detection, better disease management, and more effective public health interventions. These findings emphasize the need for tailored public health interventions to improve the diagnosis and management of EPTB, especially in vulnerable populations, by identifying high-risk group.

## Data Availability

Data will be available upon request from the corresponding author.
